# Plasma Lipid Profile Reveals Plasmalogens as Potential Biomarkers for Colon Cancer Screening

**DOI:** 10.3390/metabo10060262

**Published:** 2020-06-25

**Authors:** Anna Maria A.P. Fernandes, Marcia C.F. Messias, Gustavo H.B. Duarte, Gabrielle K.D. de Santis, Giovana C. Mecatti, Andreia M. Porcari, Michael Murgu, Ana Valéria C. Simionato, Thalita Rocha, Carlos A.R. Martinez, Patricia O. Carvalho

**Affiliations:** 1 Laboratory of Multidisciplinary Research, São Francisco University, Bragança Paulista, São Paulo 12916-900, Brazil; marcia_cfmessias@hotmail.com (M.C.F.M.); gabi.kris.98@gmail.com (G.K.D.d.S.); giovana.mecatti@usf.edu.br (G.C.M.); andreia.porcari@usf.edu.br (A.M.P.); thalita.rocha@usf.edu.br (T.R.); carlos.martinez@usf.edu.br (C.A.R.M.); patricia.carvalho@usf.edu.br (P.O.C.); 2 Institute of Chemistry (IQ), University of Campinas (UNICAMP), Campinas, São Paulo 13083-970, Brazil; gustavo_duarte95@hotmail.com; 3 Luiz Barssotti Application Laboratory, Waters Technologies from Brazil, Barueri, São Paulo 06455-020, Brazil; michael_murgu@waters.com; 4 National Institute of Science and Technology in Bioanalytics, IQ, UNICAMPCampinas, São Paulo 13083-970, Brazil; avsimionato@iqm.unicamp.br; 5 IQ, (UNICAMP), Campinas, São Paulo 13083-970, Brazil

**Keywords:** colon cancer, biomarkers, lipidomic, mass spectrometry, plasmalogens

## Abstract

In this era of precision medicine, there is an increasingly urgent need for highly sensitive tests for detecting tumors such as colon cancer (CC), a silent disease where the first symptoms may take 10–15 years to appear. Mass spectrometry-based lipidomics is an emerging tool for such clinical diagnosis. We used ultra-performance liquid chromatography coupled to electrospray ionization quadrupole time-of-flight mass spectrometry operating in high energy collision spectral acquisition mode (MS^E^) mode (UPLC-QTOF-MS^E^) and gas chromatography (GC) to investigate differences between the plasmatic lipidic composition of CC patients and control (CTR) subjects. Key enzymes in lipidic metabolism were investigated using immuno-based detection assays. Our partial least squares discriminant analysis (PLS-DA) resulted in a suitable discrimination between CTR and CC plasma samples. Forty-two statistically significant discriminating lipids were putatively identified. Ether lipids showed a prominent presence and accordingly, a decrease in glyceronephosphate O-acyltransferase (GNPAT) enzyme activity was found. A receiver operating characteristic (ROC) curve built for three plasmalogens of phosphatidylserine (PS), named PS(P-36:1), PS(P-38:3) and PS(P-40:5), presented an area under the curve (AUC) of 0.998, and sensitivity and specificity of 100 and 85.7% respectively. These results show significant differences in CC patients’ plasma lipid composition that may be useful in discriminating them from CTR individuals with a special role for plasmalogens.

## 1. Introduction

Colorectal cancer (CRC) accounts for 1 in 10 cancer cases and deaths worldwide and is the third pathology in terms of incidence, and second in terms of mortality [1,2]. Colon cancer (CC) alone is the fourth most diagnosed type of cancer, representing more than one million new cases worldwide in 2018, more than fifty percent of which resulted in death [1]. Cancers in the colon and rectum are frequently studied together; however, there are clinicopathological differences in cancers across those sites [3].

As the CC stage advances, the five-year survival rate decreases [4]. Less invasive bio-fluid-based early detection strategies (urine and peripheral blood and its components) are attractive alternatives compared to colonoscopic screening methods [5,6]. Additionally, unraveling and understanding alterations in plasma metabolic profile associated with cancer could lead to the development of new strategies for cancer cure and prevention.

Lipids are fundamental metabolites in human physiology that play vital roles in the homeostasis of the organism at the cellular level, acting as structural molecules in the cell membrane and, at a systemic level, as intra- and inter-cellular signals. Accordingly, many human diseases, including metabolic, immune, and central nervous system diseases, as well as cancer, are consequences of metabolic changes in lipid enzymes and their pathways [7,8].

Chromatographic techniques coupled to mass spectrometric methods, combined with multi and univariate statistical analyses have been extensively used in the past few decades, constituting powerful and indispensable lipidomics tools. While gas chromatography (GC) enables the qualitative and quantitative evaluation of fatty acid (FA) composition of the lipidic portion in biological samples, the use of ultra-performance liquid chromatography coupled to electrospray ionization quadrupole time-of-flight mass spectrometry operating in high energy collision spectral acquisition mode (MS^E^) mode (UPLC-QTOF-MSE) analyzer makes it possible to separate the major lipid classes with high resolution and sensitivity, which improves the detection of low-abundant lipid species [9]. Also, the high energy collision spectral acquisition mode (MS^E^) increases the amount of biological information that may be obtained from a single sample. MS^E^ scans enable simultaneous acquisition of full scan data and collision-induced fragmentation to improve the identification of lipid classes and to obtain structural information [10].

Mass spectrometry-based lipidomics studies have been applied to different types of cancer [11,12,13,14,15,16,17]. Although many other studies have reported alterations in metabolic end products for CRC [18,19,20,21,22,23,24], none of them, to the best of our knowledge, have investigated the alterations of lipids in plasma of CC patients.

In this work, we used a UPLC-QTOF-MS^E^-based untargeted lipidomic approach to investigate differences between the lipid profiles of CC patients and those of CTR volunteers. FA composition was complementarily analyzed by GC coupled to flame ionization detection (GC-FID). The objective was to identify potential biomarkers and metabolic pathways associated with CC in order to contribute to a better understanding of this pathology and to indicate putative diagnostic biomarkers. As a consequence, the key enzymes in the biosynthesis of the plasmalogens [25,26] glyceronephosphate O-acyltransferase (GNPAT), lysophosphatidylcholine acyltransferase 4 (LPCAT4) and stearoyl-CoA desaturase 1 (SCD1) were investigated by enzyme-linked immunosorbent assay (ELISA). Plasmalogens are a special class of ether lipids. It has already been shown that plasmalogen levels are much higher in cancer cells than in normal cells [27]. Those findings have encouraged many endeavors to establish plasmalogens as tumor markers in medical cancer diagnostics [28].

## 2. Results

### 2.1. Demographic and Clinicopathological Characteristics of CC and CTR Subjects

Participants in this study included 50 CC and 50 CTR volunteers, aged from 52 to 74 and 42 to 76 in CC patients and CTR volunteer groups, respectively. Among the CC patients, 58% had CC in early stages (tumor, node, metastasis classification system (TNM) staging I and II, respectively) [29], 24% had CC at an advanced stage (TNM staging III and IV), and 18% were determined as unclassified. The demographic and clinicopathological characteristics of the subjects are summarized in Table 1. Male and female subjects were equally added to the protocol. No significant differences were found in the parameters age and body mass index (BMI).

### 2.2. Untargeted Lipidomic Plasma Analysis and Discrimination Between CC Patients and CTR Volunteers

**Figure 1:**
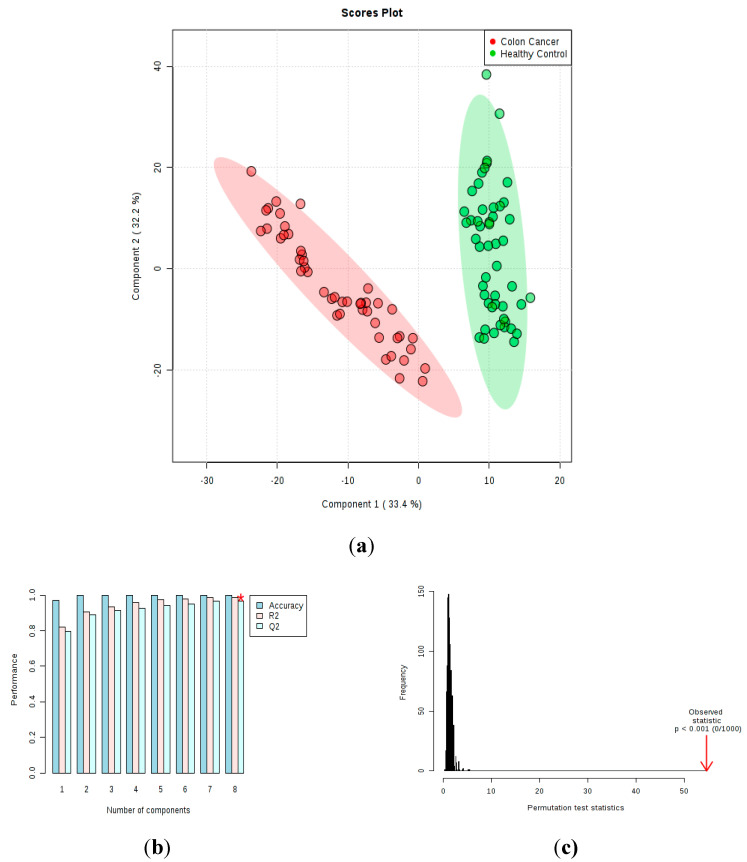
Partial least squares discriminant analysis (PLS-DA) scores plot for control (CTR) volunteers (green) and colon cancer (CC) patients (red). (**a**) As shown, 33.4% and 32.2% of the variance of the samples are explained by components 1 and 2, respectively. (**b**) Performance measures of the PLS-DA model (prediction accuracy, R2, and Q2), *best value of Q2 (0.97). (**c**) Permutation test at 1000 permutations (*p* < 0.001).

A total of 2190 features were detected in the positive and 2528 in the negative ionization modes. Compound detection was based on extracted ion chromatograms. Principal Component Analysis (PCA) was performed with all the features obtained from both ionization modes, achieving good segregation between CC and CTR groups (Figure S1). Partial least squares discriminant analysis (PLS-DA) confirmed the excellent discrimination between the CC patients and CTR volunteer groups (Figure 1a, positive mode) with good predictive performances (R2 = 0.90 and Q2 = 0.89, two components, Figure 1b) and a very significant *p*-value (<0.001) for the permutation test (Figure 1c). These findings were observed in the negative ionization mode, as well (Figure S2).

Heatmap analysis, which provides a peripheric view of intensities, samples and groups (Figure S3), demonstrates some discrimination between male and female volunteers in the CTR group which is not observed in CC patients. This tendency of segregation between male and female individuals in the CTR but not in the CC group was also found in the PCA analysis (Figure S4).

Features were then classified according to their variable importance in projection (VIP) scores in the first component, log2 fold change (log2(FC)), *p*-value, false discovery ratio (FDR) and area under the curve (AUC) of the receiver operating characteristic (ROC) plot, resulting in the selection of 167 statistically significant features in the positive ionization mode, and 141 features in the negative ionization mode. Progenesis QI identification and Human Metabolome Database (HMDB) matching resulted in the annotation of 31 out of the 167 significant features as putative biomarkers for CC plasma samples classification (Table 2 and Table S1).

**Figure 2:**
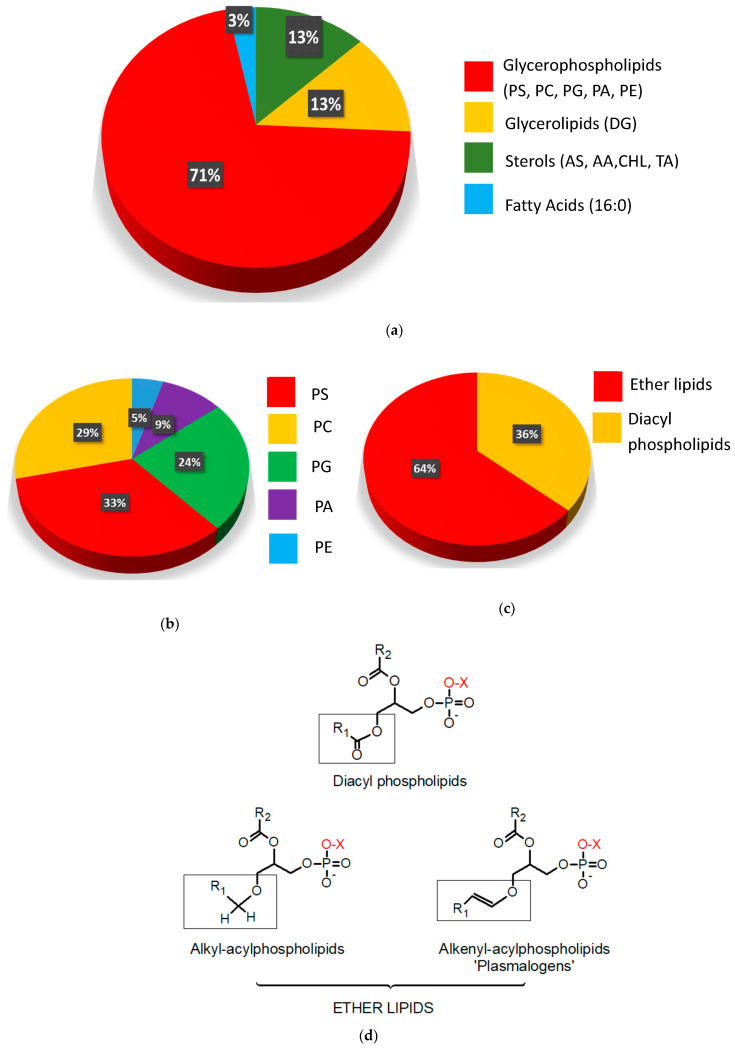
Distribution of the annotated metabolites according to major lipid classes. (**a**) AS = androsterone sulfate, AA = apocholic acid, CHL = cholesterol, TA: trihydroxycoprostanoic acid, DG: diacylglycerols and Fatty acid 16:0 = palmitic acid. (**b**) Distribution in the class of glycerophospholipids (GPL): PS: phosphatidylserine, PC: phosphatidylcholine, PG: phosphatidylglycerol, PA: phosphatidic acid and PE: phosphatidylethanolamine. (**c**) Percentages of ether and diacyl GPL. (**d**) General chemical structures of GPL where X: serine, choline, glycerol, H or ethanolamine. The different kinds of binding to the fatty acid moiety of the glycerol oxygen in the SN1 position are highlighted.

Taking data from Table 2 into account, glycerophospholipids (GPL) was, by far, the class of compounds that most contributed to the separation of groups in our study, accounting for 71% of the classifier features. It was followed by glycerolipids (13%), sterols (13%) and one fatty acid (3%) (Figure 2a). Within the GPL class, those from phosphatidylserine (PS) showed some prevalence responding for 33% of the total followed by those from phosphatidylcholine (PC, 29%), phosphatidylglycerine (PG, 24%), phosphatidic acids (PA, 9%) and phosphatidylethanolamines (PE, 5%, Figure 2b).

Ether lipids are the most prominent subclass of GPL, corresponding to 64% of the total (Figure 2c). Figure 2d shows key differences in the chemical structures of these phospholipids. Three different kinds of fatty acids (FAs) bound to the SN-1 position of the glycerol backbone, generate three different subclasses named: (i) diacyl phospholipids for an ester bond; (ii) alkyl-acylphospholipids for an ether bond; (iii) plasmalogen (or alkenyl-acylphospholipid) when there is a double bond adjacent to the oxygen in the ether group. Alkyl-acylphospholipids and plasmalogens are also known as “ether lipids” [31].

### 2.3. Phosphatidylserine Plasmalogens as Biomarkers for CC Diagnostics

**Figure 3:**
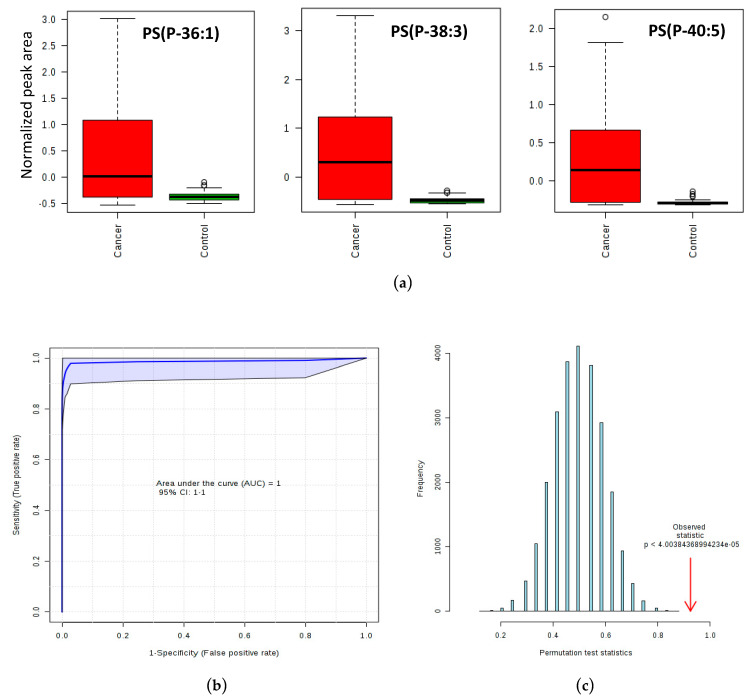
Plasmalogens elected for the predictive support vector machine (SVM) model. (**a**) Boxplots of the phosphatidylserine plasmalogens PS (P-36:1), PS (P-38:3) and PS (P-40:5). Data are represented as box plots with the median and 25th and 75th percentiles; error bars indicated the lowest and highest values. Open circles above and below the whiskers indicate outliers outside the 10th and 90th percentiles. (**b**) Receiver operating characteristic (ROC) curve (and 95% confidence interval) exhibiting an area under the curve (AUC) of 0.998 for the plasmalogens predictive model. (**c**) Permutation test result for the SVM model (*p* < 0.05).

As demonstrated in the boxplots of Figure 3a, PLS-DA analysis revealed that the phosphatidylserine plasmalogens PS (P-36:1), PS (P-38:3) and PS (P-40:5) were more abundant in CC than in CTR samples. These PS plasmalogens were then used to construct aROC curve, which plots the sensitivity (true positive rate) as a function of 1-specificity (false positive rate, for a 95% confidence interval - CI), shown in Figure 3b. The model presented an area under the curve (AUC) of 0.998, reflecting the outstanding ability of the selected plasmalogens to distinguish between CC and CTR groups. Figure 3c shows the *p*-value for the permutation test (found to be very significant). The resulting support vector machine (SVM) model was applied to classify the validation set, correctly classifying 30 out of 35 CTR samples and all cancer samples. Therefore, the positive predictive value (PPV) of these plasmalogens is 86.1%, and the negative predictive value (NPV) is 100%, with a specificity of 85.7%, and a sensitivity of 100% for a per-patient analysis. The average accuracy based on 100 cross validations was 91.1%.

### 2.4. Analyses of Fatty Acid Composition by GC

Thetypes of FA found in all groups were the saturated FA (SFA) followed by the n-6 poly-unsaturated FA (PUFA) and the mono-unsaturated FA (MUFA) (Table 3). Slight differences from CTR volunteers were observed according to the cancer stage for SFA (14:0), which was reduced in stage II, and n-6 PUFA (20:4 n-6), which was reduced in stages III/IV. Significant reductions were observed for 22:5 n-3 PUFA for CC in stages I and III/IV and 22:6 n-3 for all stages, when compared to the CTR group. No statistical differences were observed among the evaluated groups when considering total plasma FA in different cancer stages.

### 2.5. Metabolic Pathway Analyses Plot

**Figure 4:**
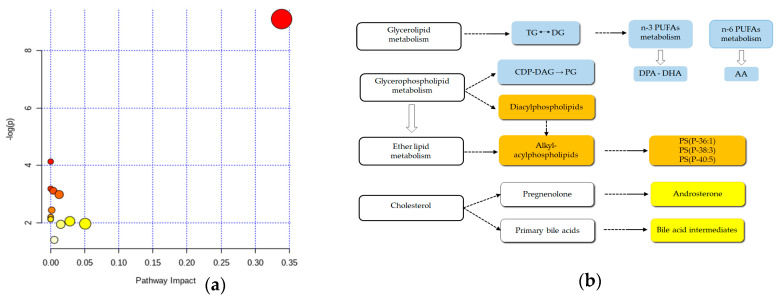
Metabolic pathways based on the identified potential biomarkers. (**a**) Pathway analysis performed using MetaboAnalyst 4.0. (*false discovery rate, FDR < 0.05). (**b**) Diagram of pathways associated with the lipid biomarkers of colon cancer CC plasma samples. Colored boxes represent an increase (orange and yellow) or decrease (blue) of metabolites when compared to (CTR) subjects. Diacylglycerols (DG), Triacylglycerols (TG), Cytidine Diphosphate-DG (CDP-DG), PUFA: polyunsaturated fatty acids, DPA: docosapentaenoic acid and DHA: docosahexaenoic acid, ARA: arachidonic acid, PS: phosphatidylserine.

Pathway analysis was performed with the differentiated metabolites (Figure 4a). The size and the position of the circles show the impact of the metabolite on the pathway. Indeed, larger circles, which are also those with higher coordinate values, show a more prominent impact of those metabolites on the respective pathway. The impact values and other statistical data of the pathway analyses are depicted in Table S2. The graph gives a visual representation of the relevance of the GPL metabolism pathway for the differentiation of CC and CTR plasma samples when considering our panel of metabolites. The graph also shows that other pathways, such as that of primary bile acids biosynthesis and glycerolipids, were also impacted although without statistical significance at this point. Figure 4b shows an integrated diagram of lipid metabolic pathways, including ether lipid metabolism, bile acids biosynthesis, glycerolipids metabolism, n-6 and n-3 PUFA.

### 2.6. GNPAT, SCD and LPCAT4 Concentrations in Plasma as Determined by Enzyme-Linked Immunosorbent Assay (ELISA) Assay

**Figure 5:**
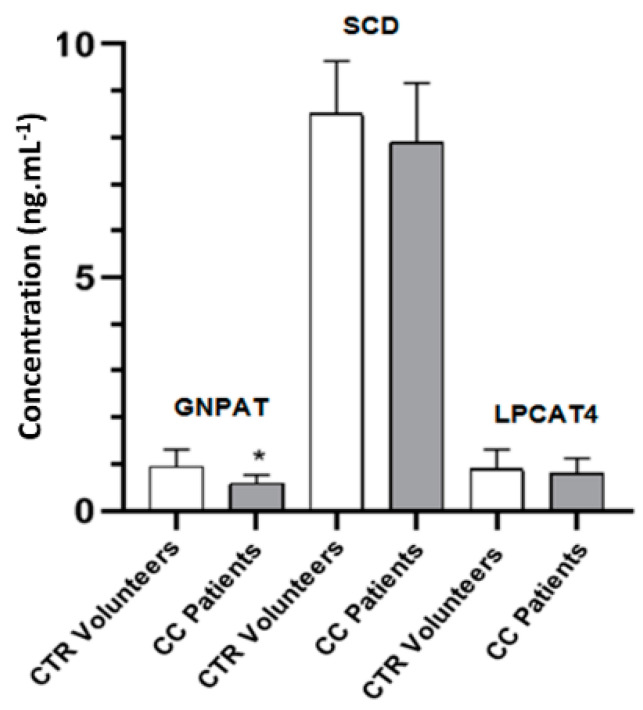
In vitro quantitative measurement of plasmalogen synthesis enzymes. Glyceronephosphate O-acyltransferase (GNPAT), stearoyl-CoA desaturase 1 (SCD) and lysophosphatidylcholine acyltransferase 4 (LPCAT4) concentrations in plasma samples from colon cancer (CC) patients and control (CTR) volunteers. Values expressed as mean ± standard deviation * *p* < 0.05.

Concentrations of GNPAT, SCD, and LPCAT4 in plasma samples from CC patients and CTR volunteers are described in Figure 5. ELISA analysis revealed a decrease in the concentration of GNPAT in the plasma of CC patients when compared to CTR volunteers (*p* < 0.05). Regarding the concentrations of SCD and LPCAT4, no statistical difference was observed between the groups.

## 3. Discussion

This study reports the lipidomic investigation of plasma samples from CC patients compared to CTR volunteers in order to detect putative lipid biomarkers with the potential to differentiate both groups. Fatty acid and enzyme analyses were also used to complement the findings and assist in the comprehension of the metabolic pathways most impacted by the carcinogenesis process. UPLC-QTOF-MS^E^ data assisted by multivariate analysis showed a clear difference between the plasma lipid profile of CC patients and CTR volunteers and 31 compounds were revealed as the most relevant and statistically significant molecules for discriminating the groups. We found that gender causes some segregation in the CTR group but not in CC patients pointing to the observation that the cancer state causes more relevant changes in the lipidic profile of the subjects than gender.

In the MS^E^ mode of acquisition, MS and tandem mass spectrometry (MS/MS) data are acquired from the same single analytical run. Alternating scans are acquired at either low or high collision energies in the collision cell, thus producing precursor ions and fragments information. This technique improves the efficiency of the instrument in terms of the amount of data produced since all analytes are fragmented without the need of a pre-selection of an analyte m/z value in the quadrupole [32].

For Liquid chromatographic mass spectrometry (LC-MS) analysis and the building of the predictive SVM model, the results from both positive and negative ionization modes were used. From the three features included in the predictive model, one plasmalogen, PS (P-40:5), was exclusively detected in negative ion mode (Table S1). Indeed, negative ion mode enabled the detection of the majority of the features included in Table 2, suggesting this ion mode should be privileged for target approaches.

GPL was found to be the most relevant category of lipids in this study and, among the subcategories, PS was the one that contributed most to our list of differential features. PS is an immunosuppressive anionic phospholipid whose essential functions are to activate important kinases, such as protein kinase C (PKC), 3-Phosphoinositide-dependent kinase 1 (PDK1) and protein kinase B (AkT), and it serves as an interaction molecule for several signaling proteins [33,34]. The process of tumorigenesis involving PS occurs because cancer cells inhibit the maturation of dendritic cells and decrease the production of cytotoxic T cells [35]. Overexpression of PS has already been observed in human breast cancer cell lines (MDA-MB-231-Luc-D3H2LN), glioblastoma (Gli36) and astrocytoma (U371), and CRC [34].

It is also worth noting the relative amount of ether lipids among the list of potential biomarkers of CC, specially GPL. In humans, the average concentration of ether lipids is 20% of the pool of the phospholipids, varying according to the tissue. In this work, more than 60% of the pool of GPL were ether lipids [36].

Higher levels of ether lipids in tumors have been described since the late 1960s [37,38] and since then, many reports have correlated these molecules to pathological states such as breast cancer [27] and to anti-metastatic drug’s action mechanisms [39].

Plasmalogens can be considered as a subset of ether lipids [40]. The ROC curve, constructed to evaluate the potential of PS plasmalogens as potential diagnostic biomarkers, showed excellent sensitivity, 100% (NPV of 100%), meaning that all cancer subjects would be positively classified as cancer in our predictive model. Additionally, a blood test using this panel of biomarkers could possibly be used before colonoscopy, as confirmed by the specificity achieved by this model (PPV of 86.1%). These results are in agreement with previous studies that also point the plasmalogens as good candidates for biomarkers of cancer disease [41]. However, a higher number of samples must be analyzed before that, in order to biologically validate the biomarker characteristics of these plasmalogens. These preliminary results are comparable to, or even better than other non-invasive and non-radiologic biofluid-based screening methods for CRC, especially in terms of sensitivity. The Food and Drug Administration (FDA)-approved fecal immunochemical test combined with stool DNA test (FIT-DNA) showed a sensitivity for detection of CRC of 92.3% (specificity of 86.6%), while the blood-based analysis for the presence of circulating methylated SEPT9 DNA has shown a sensitivity of 48.2% and a specificity of 91.5% [42].

Androsterone sulfate (AS), apocholic acid (AA), cholesterol (CHL) and trihydroxycoprostanoic acid (TA) are sterols [7] and were found in increased amounts in plasma samples from CC patients. Statins, the first choice medication to control the low-density lipoprotein (LDL) cholesterol levels in the blood, have been successfully tested in CRC patients [43] and in CC stem cells [44] suggesting the participation of this metabolite in cancer progression. Trihydroxycoprostanic acid is a C27-bile acid intermediate, which is converted to cholic acid in the peroxisomes [45,46]. Bile acids are steroid acids primarily produced by the liver and metabolized by enteric bacteria in the colon to form secondary toxic derivatives that have been implicated in the acceleration and progression of CRC [47,48]. Recently, a consistent increase in genes for secondary bile acid conversion in CRC-associated microbiomes has been reported [49]. Our pathway analysis also reported primary bile acid biosynthesis as one of the impacted pathways, albeit without statistical significance at this point. Our results are in agreement with the connection between a fat/meat-rich diet and CC occurrence hypothesis [48]. More specific experiments must be performed to better explore these results. Androsterone sulfate is a constituent of the sulfated sterol fraction of the human blood and the most abundant 5α-androgen [50,51]. It has been suggested that the combined levels of androsterone sulfate and epiandrosterone sulfate (EpiA-S) could be one of the markers of the 5α-reductase activity, an enzyme related to androgen-dependent diseases, such as prostate cancer [52]. To the best of our knowledge, this is the first time this metabolite has been implicated in CC.

Samples analyses by UPLC-QTOF-MS^E^ showed lower relative concentration of palmitic acid in plasma samples of CC patients when compared to normal control volunteers. The depletion of palmitic acid levels has been previously observed in CC plasma samples [17,53]. Palmitic acid is a key intermediate in the biosynthesis of FA. The tumor microenvironment is extremely flexible in its metabolic demands and tumor cells may become dependent on saturated FA uptake during oxygen restrictions and unsaturation impairment [54].

GC-FID analysis elucidated more information about FA variations according to CC stage. The results showed a reduction in the 22:6 n-3 PUFA levels in CC, suggesting that the synthesis of PUFA —and possibly its oxidation products—have a prominent role in CC. One possible explanation is the high susceptibility of PUFAs to oxidation due to the presence of multiple double bonds in them, as previously reported by our group for patients with rectal adenocarcinoma [17]. Lipid peroxidation with the formation of reactive compounds, such as malonaldehyde, hexanal, and 4-hydroxynonenal, leads to changes in the permeability and fluidity of the membrane lipid bilayer altering cell integrity [55] and has been described as an important determinant of cancer cell function [56,57]. Preliminary data have also reported lower 3-PUFA content in plasma samples of CRC [58] and rectal adenocarcinoma patients [17], as well as a decrease in very-long-chain dicarboxylic acid 28:4 in plasma levels from Italian and Brazilian CRC patient cohorts [59].

An increased relative abundance of PE (32:2) in CC samples was also observed in this study. PAs showed a dual tendency. While PAs formed by saturated FA presented an increase in their relative abundance in CC samples, those PAs with unsaturated FA (20:3 and 20:4) were lower in these samples. These findings are in good agreement with our GC analysis which showed a significant decrease in the arachidonic acid (20:4 n-6) levels, especially at III/IV cancer stages. PGs were only detected in unsaturated form ((PG(38:5), PG(38:6) and PG(38:7)) and in decreased concentrations in CC patients, in agreement with what was found in colorectal tissue [60].

Recently, by employing UHPLC-Q-TOF MS and UHPLC-QQQ MS platforms, 11 lipid species including PE plasmalogens (PE(P-18:2/18:2), PE(P-18:1/18:2), PE(P-18:1/22:5)) and fatty acids (20:0, 20:5, 22:4) were identified as discriminators of CRC patients at an early stage from healthy controls [61].

Enzymatic analysis showed a significant reduction of GNPAT concentration as well as a non-significant reduction of SCD in CC patients compared to CTR individuals. In the biosynthesis of ether lipids, the peroxisomal enzymes, GNPAT and AGPS, generate 1-alkyl dihydroxyacetophosphate (1-alkylDHAP) by replacing the acyl chain of 1-acyl-DHAP with a fatty alcohol that is synthesized by fatty acyl-CoA reductase 1 (FAR1) [36,62]. Considering that LPCAT4, SCD, and GNPAT are all intracellular enzymes and were quantified in the plasma, that may have made the assessment of the real interference of those enzymes in the alteration of the plasmalogens more difficult. Experimental evidence suggests that the rate-limiting step of ether lipids synthesis is that of fatty alcohol synthesis by FAR1 that is subject to feedback regulation by cellular plasmalogen levels which can induce FAR1 protein degradation [63]. Those results underscore the need for more specific studies of the action of those enzymes in the altered metabolic pathways related to plasmalogen synthesis in colon cancer patients.

## 4. Materials and Methods 

### 4.1. Reagents

High performance liquid chromatography (HPLC) grade acetonitrile (ACN) and isopropanol were from Honeywell (Morristown, NJ, USA) and methanol, formic acid and ammonium formate from Sigma-Aldrich (Saint Louis, MO, USA). Chloroform and hexane were from Merck (Darmstadt, HE, Germany). Water was purified on a Milli-Q system from Millipore (Medford, MA, USA). Antibodies and their respective sources were as follows: human LPCAT4, GNPAT and SCD were purchased from Cloud-Clone Corp^®^ (Miami, FL, USA).

### 4.2. Volunteers, Ethical Consent and Plasma Samples

This cross-sectional study included fifty healthy volunteers from the CTR group and fifty CC patients from the São Francisco University Hospital (HUSF). Patient recruitment took place from January 2018 to June 2019. Before blood collection, written informed consent was acquired from all the participants and the same protocol was applied to both CC and CTR volunteers. The study was approved by the ethics committee of the São Francisco University, CAAE 57114716.8.1001.5514. Venous blood was collected from 12 hour-fasted individuals using sampling tubes with potassium ethylenediamine tetraacetic acid (EDTA) anticoagulant. After collection, the blood was centrifuged for 10 min at 2500× *g* at 20 °C and the plasma was fractionated into 100 μL aliquots in micro centrifuge tubes and immediately stored at −80 °C until analysis. For CC patients, samples were taken prior to their submission to surgical procedures, chemotherapy, and/or radiotherapy. Only CC patients with diagnoses confirmed by histopathology were considered. The Tumour, Node, Metastasis (TNM) classification system of the American Joint Committee on Cancers (AJCC) [29] was used to stratify the CC patients into three groups for analysis: stage I, stage II and stage III/IV. Only non-smoker CTR volunteers were selected and screened based on hemato-biochemical analysis combined with clinical examination.

### 4.3. Total Lipids Extraction

Plasma samples (0.8 mL) were extracted with 2.5 mL of chloroform–methanol (2:1) and 0.5 mL of an aqueous solution of NaCl (0.1 M) as previously reported by Folch et al. [64]. The lower organic layer was collected and separated into two fractions that were dried under nitrogen flow and stored at −80 °C for analysis within a period of less than 6 months [65,66].

### 4.4. Lipid Profile of Plasma Samples by UPLC-QTOF-MS^E^ Analysis

For UPLC-QTOF-MS^E^ analysis, dried lipid samples were reconstituted in 1 mL of an isopropanol/acetonitrile/water (2:1:1, *v*/*v*/*v*) solution. Data were acquired using an ACQUITY FTN liquid chromatograph coupled to a XEVO-G2XSQTOF mass spectrometer (Waters, Milford, MA, USA) using MassLynx 4.1 software and the column used was an Acquity UPLC CSH C18 column (2.1 × 100 mm, 1.7 μm, Waters). The mobile phase consisted of 10 mM of ammonium formate with 0.1% formic acid in acetonitrile/water (60:40, *v*/*v*) (A) and 10 mM ammonium formate with 0.1% formic acid in isopropanol/acetonitrile (90:10, *v*/*v*) (B) at a flow rate of 0.4 mL min-1 with a linear gradient (in % B): 0–0.5 min: 40–43%; 0.5–0.6 min: 63%; 0.6–4 min: 68%; 4–4.1 min: 70%; 4.1–6.5 min: 99%; 6.5 min: decrease to 40%; 6.5–8.1 min: 40% (with a further 1.9 min for column reequilibration) resulting in a 10 min analysis. The injection volume was 0.1 µL. For the electrospray ionization source, the parameters were set as follows: capillary voltage of 2 kV, sampling cone of 30 V, source temperature of 130 °C, desolvation temperature of 450 °C, cone gas flow of 100 L·h^−1^, desolvation gas flow of 800 L·h^−1^ for positive mode; capillary voltage of 1.0 V, sampling cone of 40 V, source temperature of 130 °C, desolvation temperature of 450 °C, cone gas flow of 50 L·h^−1^, desolvation gas flow of 800 L·h^−1^ for negative mode. The acquisition scan range was from 50 to 2000 Da and the data were acquired using an MS^E^ approach. Leucine encephalin (molecular weight = 555.62; 200 pg·μL^−1^ in 1:1 ACN:H2O) was used as a lock mass for accurate mass measurements, and a 0.5 mM sodium formate solution was used for instrument calibration. Pooled samples were injected every twenty injections. Raw data were deposited in the MetaboLights data repository (study code MTBLS1584).

### 4.5. Fatty Acid Profile by GC-FID Analysis

Dry lipid extracts were treated with boron trifluoride-methanol for FA derivatization before GC analysis. The fatty acid methyl esters (FAME) resuspended in hexane were injected in the splitless mode (1 μL) and analyzed by GC in triplicate, using a CP 9001 GC-FID chromatograph (CHROMPACK, Middelburgburg, ZE, Netherlands) and a capillary column CP-Sil 88 (WCOT Fused Silica 59 m × 0.25 mm) as previously reported [17]. FA identification was performed by comparing the retention time of sample components with authentic FAME standards (Supelco Chemical Co., Bellefonte, PA, USA) injected under the same conditions. FA composition was expressed in relation to the percentage of total FA and calculated according to the area value of each peak using the Chromatostation N2000 system (Surwit Technology Inc., Hangzhou, ZJ, China). GC-FID data were expressed as mean ± standard deviation (SD).

### 4.6. Immunodetection of Plasmalogen Synthesis Enzymes LPCAT4, SCD and GNPAT

In vitro quantitative measurements of LPCAT4 (SEG532Hu 96 Tests), SCD (SEF419Hu 96 Tests) and GNPAT (Cat.No: BS9320649) in plasma samples were carried out using commercial quantitative sandwich ELISA kits (Cloud-Clone Corp^®^, Miami, FL, USA for LPCAT4 and SCD; MyBioSource.com, San Diego, CA, USA for GNPAT) according to the manufacturer’s instructions. ELISA data were analyzed using a Stat Fax 2100 reader (Awareness Technology, Palm City, FL, USA). The values were processed automatically by the program MultCalc (PerkinElmer Life Sciences, Waltham, MA, USA). The analysis was performed in triplicate and for the mean and standard deviation calculations, the numerical values of the blank, standards, controls and plasma samples were considered. Results were expressed in ng·mL^−1^.

### 4.7. Data Processing, Statistical, Biomarker and Pathway Analyses

LC-MS raw data were processed with Progenesis QI software (Waters) for peak detection, alignment, integration, deconvolution, data filtering, ion annotation and MS^E^ based putative identification of compounds. Processed data are available as Supplementary Materials, namely, Spreadsheet 1: USF Colon LCMS negative processed and Spreadsheet 2: USF Colon LCMS positive processed for negative and positive modes, respectively. The LIPID MAPS [67] database was used for this identification with the following search parameters: precursor mass error ≤ 5 ppm, fragment tolerance ≤ 10 ppm. Fragmentation score, mass accuracy, isotope similarity and the Human Metabolome Database (HMDB) matching [68] were considered for the putative identification of the molecules [30]. Statistical, biomarker and pathway analyses were performed using the MetaboAnalyst 4.0 web platform [69]. Univariate and multivariate statistical analyses were carried out to find significant differences between plasma lipid profiles from CC patients and CTR volunteers. Data were normalized by sum and Pareto scaled before performing statistics. Fold change (FC), T-test and Volcano plot (Figures S5 and S6) methods were applied for univariate analysis. Only features that fulfilled log2(FC) > 1, *p*-value < 0.05 and FDR < 0.05 were considered significant. PCA was used for unsupervised, multivariate data analysis, and PLS-DA for supervised multivariate data analysis. The PLS-DA model was built using all features and a subsequent model was evaluated by cross validation and permutation tests. VIP from PLS-DA was used in addition to hierarchical analysis to construct a heat map. Classical univariate ROC analysis was performed to evaluate the linear-SVM built with the PS. The ROC curves were generated by sub-sampling where data from positive and negative modes were divided into a training set (70% of samples) and then used to build the classification model which was validated on the 30% of the samples that were left out. For GC-FID and imunoassays data, unpaired Student’s t-test and one-way analysis of variance (ANOVA) followed by Tukey’s post hoc test were performed using GraphPad Instat 3.0 and Prism 8 (GraphPad Software, La Jolla, CA, USA) respectively. A significance level of *p* < 0.05 was adopted.

## 5. Conclusions

The UPLC-QTOF-MS^E^-based untargeted lipidomic study presented here demonstrates a remarkable differentiation between the lipid composition of plasmatic samples from CC and CTR volunteers. The alterations described here are in good agreement with those previously described for CRC and other types of cancer, reinforcing the relevance of our findings. Additionally, our prediction model based on plasmalogens of PS indicates that these ether lipids can be potential biomarkers of this neoplasia and could find some application in routine screening for colorectal neoplasia. The participation of the bile acids metabolisms seems to be an interesting pathway to be further investigated. The understanding of alterations in plasmatic lipidomic profiles and metabolic pathways involved with the genesis and grown of tumors could lead to the discovery of new approaches for diagnosis, prevention or treatment of CC.

## Figures and Tables

**Table 1:** Demographic and clinicopathologic characteristics of colon cancer (CC) patients and control (CTR) volunteers.

	CC Patients	CTR Volunteers
Number of patients (N)	50	50
Sex (Male/Female)	24/26	25/25
Age (years)	62.4 ± 9.4	57.2 ± 12.9 †
BMI (kg.m^−2^)	23.9 ± 6.1	29.2 ± 6.7 †
Stages (%)		
I	11 (22)	-
II	18 (36)	-
III/IV	12 (24)	-
No information	9 (18)	-

† *p* > 0.5, body mass index (BMI). Age and BMI values expressed as mean + standard deviation.

**Table 2:** Differential features between colon cancer (CC) patients and control (CTR) volunteers.

Number	Compound ^a,b^	Molecular Formula	FDR ^c^	Mass Error (ppm)	log2(FC) ^d^	Trend
1	Androsterone sulfate	C19H30O5S	1.26 × 10^−04^	−2.42	2.25	High
2	Apocholic acid	C24H38O4	5.18 × 10^−17^	−0.76	1.14	High
3	Cholesterol	C27H46O	7.82 × 10^−11^	−0.12	5.00	High
4	TA	C13H24O2	1.64 × 10^−20^	−0.28	1.45	High
5	PA(32:0)	C35H69O8P	4.36 × 10^−23^	3.79	4.25	High
6	PA(O-34:0)	C37H75O7P	1.26 × 10^−04^	−3.61	2.23	High
7	PE(32:2)	C37H70NO8P	5.18 × 10^−17^	−3.61	1.61	High
8	PG(P-40:5) or	C46H81O9P	7.82 × 10^−11^	−0.72	3.94	High
	PG(O-38:6)					
9	PS(O-38:1)	C46H84NO9P	1.33 × 10^−10^	−2.01	4.06	High
10	PS(40:4)	C46H82NO10P	9.54 × 10^−16^	−2.92	2.46	High
11	PS(O-40:3)	C50H85NO7	4.15 × 10^−11^	−2.52	5.76	High
12N	PS(P-36:1)	C42H80NO9P	2.31 × 10^−07^	−1.42	2.49	High
12P	PS(P-36:1)	C42H80NO9P	2.69 × 10^−10^	−1.42	4.46	High
13	PS(P-38:1)	C46H84NO10P	1.74 × 10^−11^	1.81	3.91	High
14N	PS(P-38:3)	C44H80NO9P	7.34 × 10^−09^	−1.72	3.56	High
14P	PS(P-38:3)	C44H80NO9P	7.48 × 10^−10^	−1.22	4.61	High
15	PS(P-40:5)	C46H80NO9P	1.80 × 10^−08^	−2.14	4.30	High
16	DG(38:1)	C31H58O5	2.91 × 10^−27^	4.46	3.68	High
17	DG(42:5)	C45H78O5	1.78 × 10^−28^	−2.90	2.23	High
18	DG(32:1)	C35H66O5	2.01 × 10^−21^	−2.89	−1.80	Low
19	DG(34:2)	C37H68O5	1.01 × 10^−16^	−0.91	−1.09	Low
20	PA(20:3)	C23H30O6	5.37 × 10^−14^	−2.22	−1.40	Low
21	PA(20:4)	C23H39O7P	2.27 × 10^−14^	−0.97	−2.15	Low
22	Palmitic acid	C16H32O2	1.00 × 10^−15^	2.73	−1.27	Low
23	PC(36:4)	C44H80NO8P	5.71 × 10^−07^	0.41	−1.54	Low
24	PC(O-34:3)	C42H80NO7P	4.16 × 10^−10^	−1.35	−1.26	Low
25	PC(P-36:4) or	C44H80NO7P	2.52 × 10^−11^	−0.81	−1.31	Low
	PC(O-36:5)					
26	PC(P-36:3) or	C44H82NO7P	1.63 × 10^−14^	−1.06	−1.38	Low
	PC(O36:4)					
27	PC(P-38:3) or	C46H86NO7P	6.20 × 10^−11^	−4.35	−1.39	Low
	PC(O-38:4)					
28	PC(P-38:4) or	C46H84NO7P	5.13 × 10^−13^	−1.74	−1.26	Low
	PC(O-38:5)					
29	PG(38:5)	C44H77O10P	7.38 × 10^−13^	−0.76	−2.78	Low
30	PG(38:6)	C44H75O10P	1.09 × 10^−19^	3.96	−2.65	Low
31	PG(38:7)	C44H73O10P	4.18 × 10^−18^	3.81	−2.85	Low

^a.^ In parenthesis: carbon number/number of double bonds and letters P or O s for the ether lipids. ^b.^ All listed compounds reached Metabolomics Standards Initiative (MSI) level 2 identification [30], except for PE(32:2), PA(20:3), PA(20:4), PC(36:4), PG(38:5), PG(38:6), PG(38:7) and trihydroxycoprostanoic acid (TA), which reached level 3. ^c.^ FDR = False Discovery Ratio.^d.^ FC = Fold Change. N: negative mode, P: positive mode, DG= diacylglycerols, PS = phosphatidylserine, PC = phosphatidylcholine, PG = phosphatidylglycerol, PA = phosphatidic acid and PE = phosphatidylethanolamine.

**Table 3:** Fatty acid (FA) composition (relative %) of plasma total lipids in control (CTR) volunteers and colon cancer (CC) patients at different cancer stages.

Fatty Acids	CTR Volunteers	Stage I	Stage II	Stage III/IV
14:0	3.06 ± 1.63	2.08 ± 0.25	1.64 ± 0.88 *	4.51 ± 2.43
16:0	27.33 ± 4.37	30.23 ± 2.00	30.63 ± 5.79	33.25 ± 3.31
18:0	7.68 ± 1.44	7.39 ± 0.88	7.62 ± 1.70	7.38 ± 0.92
∑ SFA	38.07 ± 7.44	39.70 ± 3.13	39.89 ± 8.37	45.14 ± 6.66
16:1 n-7 (palmitoleic acid)	5.08 ± 0.79	4.40 ± 0.81	5.35 ± 2.99	5.50 ± 1.22
18:1 n-9 (oleic acid)	20.06 ± 3.36	23.04 ± 1.02	21.25 ± 5.26	19.81 ± 0.88
∑ MUFA	25.14 ± 4.15	27.44 ± 1.83	26.60 ± 8.25	25.31 ± 2.10
18:2 n-6 (linoleic acid)	28.35 ± 5.66	27.96 ± 3.41	27.77 ± 5.75	25.46 ± 1.44
20:4 n-6 (ARA)	5.12 ± 1.01	3.87 ± 2.69	4.42 ± 2.21	3.33 ± 0.62 *
∑ n-6 PUFA	33.47 ± 6.67	31.83 ± 6.10	32.19 ± 7.96	28.79 ± 2.06
18:3 n-3 (linolenic acid)	1.35 ± 1.38	0.30 ± 0.23	0.63 ± 0.32	0.42 ± 0.31
20:5 n-3 (EPA)	0.54 ± 0.42	0.35 ± 0.08	0.18 ± 0.09	0.21 ± 0.12
22:5 n-3 (DPA)	0.33 ± 0.19	0.09 ± 0.04 *	0.19 ± 0.13	0.18 ± 0.01 *
22:6 n-3 (DHA)	0.63 ± 0.28	0.22 ± 0.15 *	0.28 ± 0.07 *	0.18 ± 0.04 *
∑ n-3 PUFA	2.85 ± 2.27	0.96 ± 0.32	1.30 ± 0.54	0.98 ± 0.47

* *p* < 0.05 compared to the CTR group. Values expressed as mean ± standard deviation. SFA: saturated fatty acids. MUFA: monounsaturated fatty acids. PUFA: polyunsaturated fatty acids. EPA: eicosapentaenoic acid, DPA: docosapentaenoic acid and DHA: docosahexaenoic acid. ARA: arachidonic acid.

## Supplementary Materials

The following are available online at https://www.mdpi.com/2218-1989/10/6/262/s1, Figure S1: PCA scores plot for plasma samples. Figure S2: PLS-DA scores plots in the negative ion mode of CTR volunteers (green) and CC patients (red), Figure S3: Hierarchical clustering of samples shown as a heatmap. Figure S4: PCA scores plot for plasma samples. Figure S5: Volcano plot in the negative mode. Figure S6: Volcano plot in the positive mode. Table S1: Statistical data of differential features between CC patients and CTR volunteers. Table S2: Pathway Analysis results. Spreadsheet_1: USF_Colon_LCMS_negative_processed. Spreadsheet_2: USF_Colon_LCMS_positive_processed.

## Abbreviations

Colon cancer (CC), ultra-performance liquid chromatography coupled with electrospray ionization quadrupole time-of-flight mass spectrometry operating in MSE mode (UPLC-QTOF-MSE), gas chromatography (GC), control (CTR), partial least squares discriminant analysis (PLS-DA), glyceronephosphate O-acyltransferase (GNPAT), receiver operating characteristic (ROC), phosphatidylserine (PS), area under the curve (AUC), Colorectal cancer (CRC), fatty acid (FA), ultra- performance liquid chromatograph coupled to a quadrupole time-of-flight mass spectrometry (UPLC-QTOF), glyceronephosphate O-acyltransferase (GNPAT), lysophosphatidylcholine acyltransferase 4 (LPCAT4), stearoyl-CoA desaturase 1 (SCD1), GC coupled to flame ionization detection (GC-FID), enzyme-linked immunosorbent assay (ELISA), body mass index (BMI), Principal Component Analysis (PCA), The Tumor, Node, Metastasis classification system (TNM), American Joint Committee on Cancers (AJCC), variable importance in projection (VIP), log2 fold change (log2(FC)), false discovery ratio (FDR), Human Metabolome Database (HMDB), Diacylglycerols (DG), Phosphatidylserine (PS), Phosphatidylcholine (PC), Phosphatidylglycerol (PG), phosphatidic acid (PA), phosphatidylethanolamine (PE), glycerophospholipids (GPL), support vector machine, (SVM), positive predictive value (PPV), negative predictive value (NPV), saturated FA (SFA), n-6 poly-unsaturated FA (PUFA), mono-unsaturated FA (MUFA), docosapentaenoic acid (DPA), docosahexaenoic acid (DHA), arachidonic acid (ARA), high energy collision spectral acquisition mode (MS^E^), mass spectrometry (MS), tandem mass spectrometry (MS/MS), 1-alkyl dihydroxyacetophosphate (1-alkylDHAP), acetonitrile (ACN), fatty acid methyl esters (FAME), Androsterone sulfate (AS), apocholic acid (AA), cholesterol (CHL) and trihydroxycoprostanoic acid (TA), Ultra High Performance Liquid Chromatography coupled to quadrupole time-of-flight mass spectrometry (UHPLC-QQQ MS), Triacylglycerols (TG), Cytidine Diphosphate -DG (CDP-DG), Liquid chromatographic mass spectrometry (LC-MS), Alkylglycerone phosphate synthase (AGPS).
