# Supplementary figures and images for: Plasma Lipid Profile Reveals Plasmalogens as Potential Biomarkers for Colon Cancer Screening
Source: Metabolites. 2020 Jun 25;10(6):262. doi: 10.3390/metabo10060262 (PMC7345851; doi:10.3390/metabo10060262)

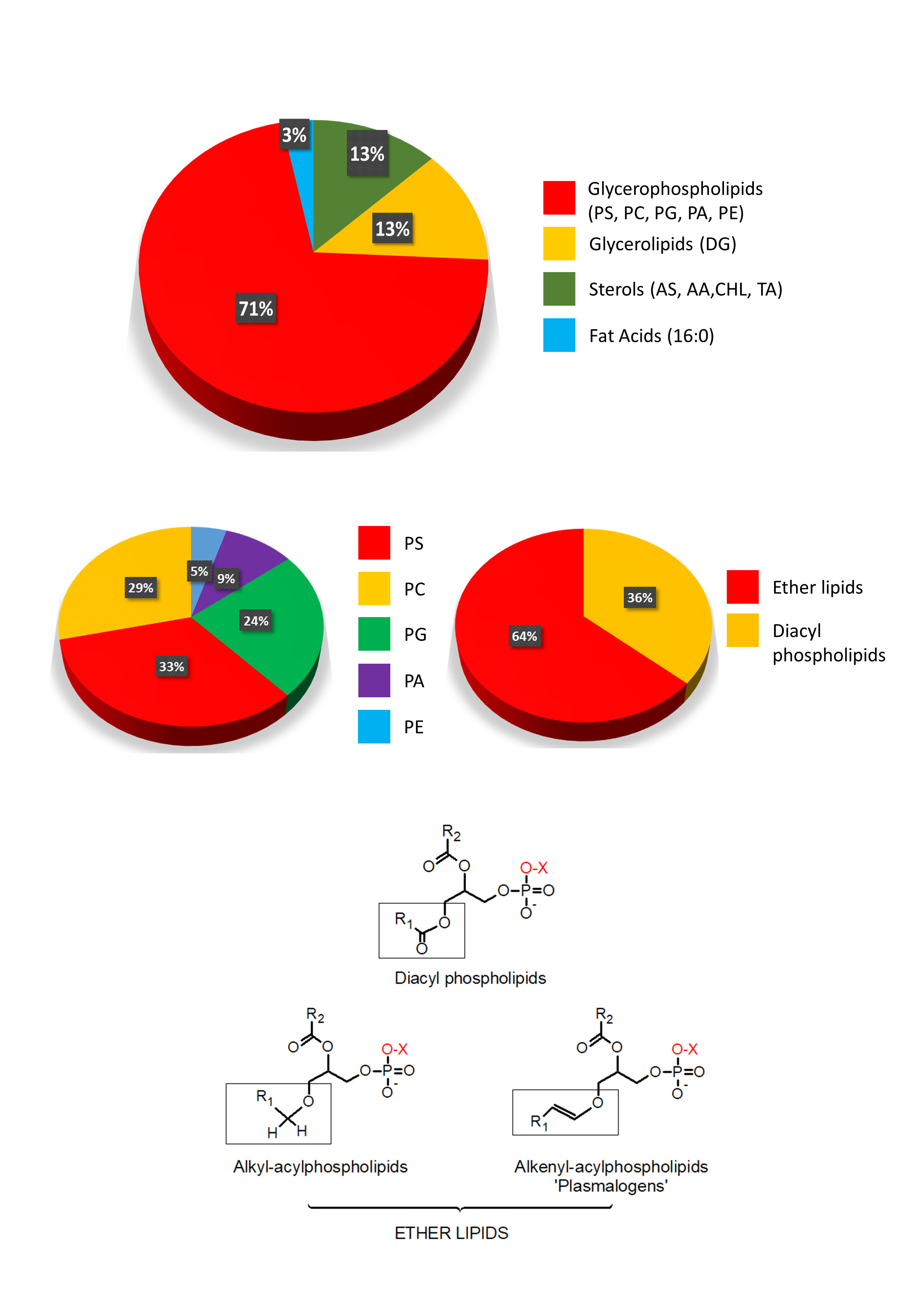

Supplement: Supplementary file 1 [file metabolites-10-00262-s001.zip › Revised_Material-826809/Figure 2_Metabolites_Revised.tif]

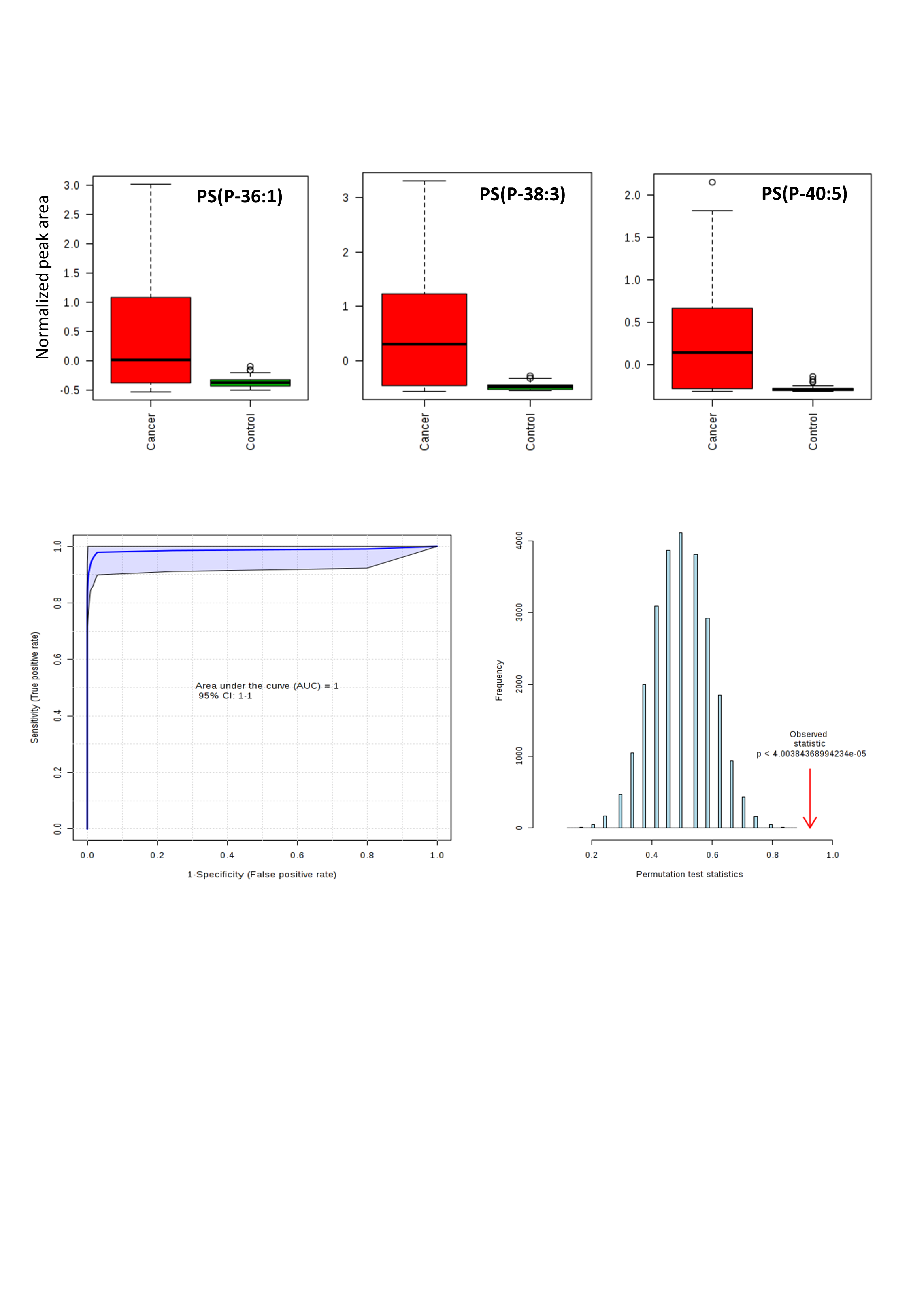

Supplement: Supplementary file 1 [file metabolites-10-00262-s001.zip › Revised_Material-826809/Figure 3_Metabolites_Revised.tif]

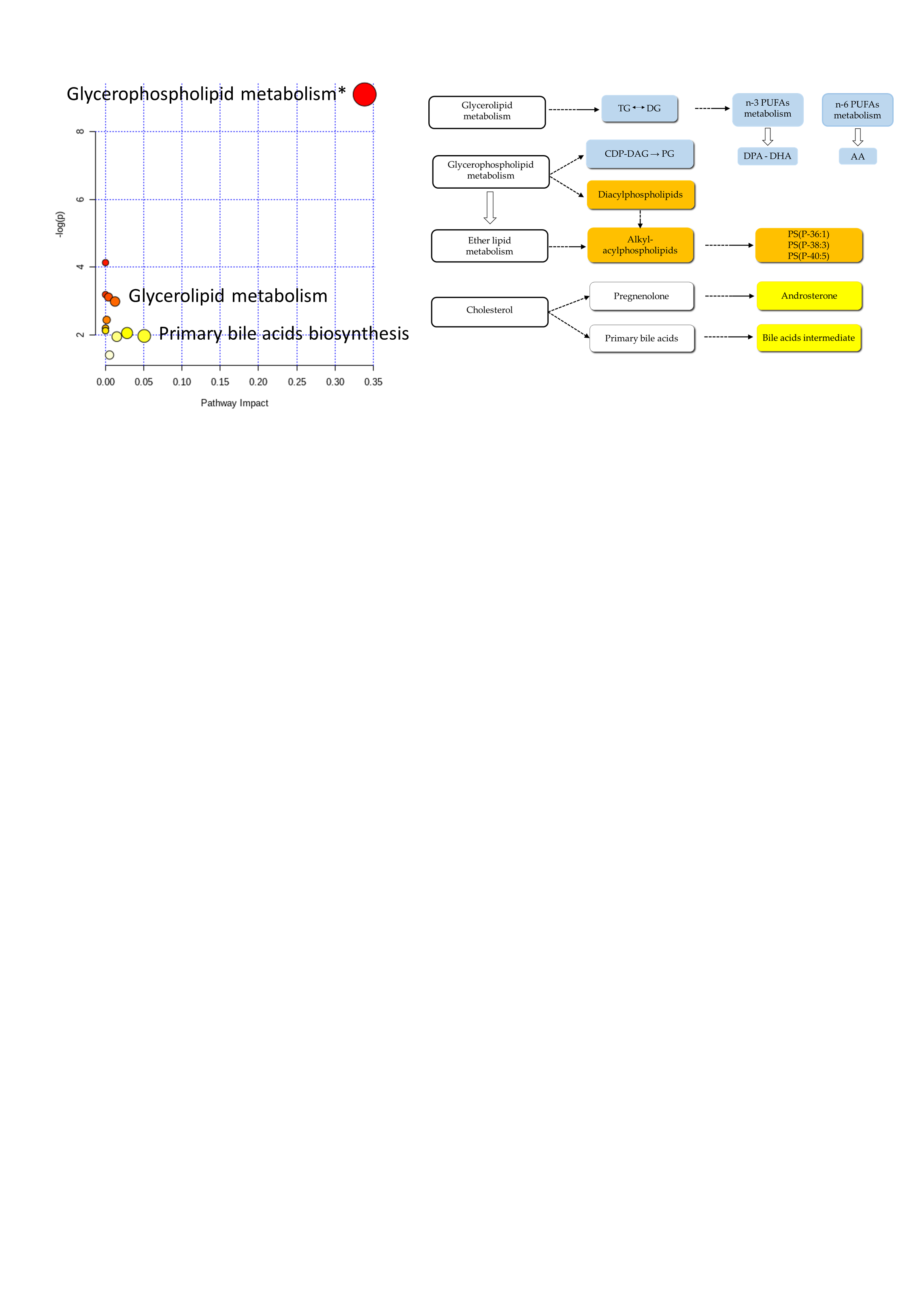

Supplement: Supplementary file 1 [file metabolites-10-00262-s001.zip › Revised_Material-826809/Figure 4_Metabolites_Revised.tif]

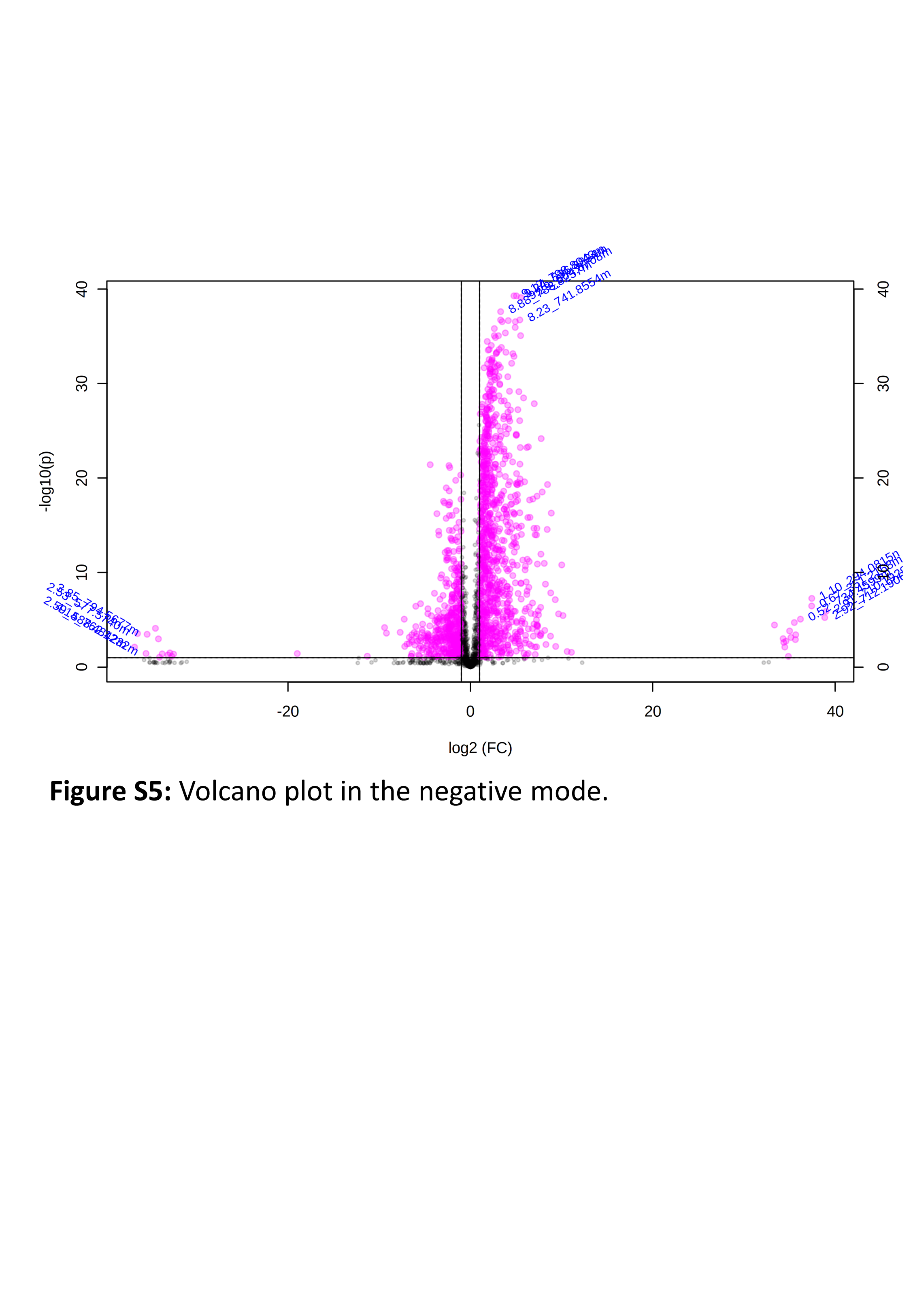

Supplement: Supplementary file 1 [file metabolites-10-00262-s001.zip › Revised_Material-826809/Figure S5_Metabolites.tif]

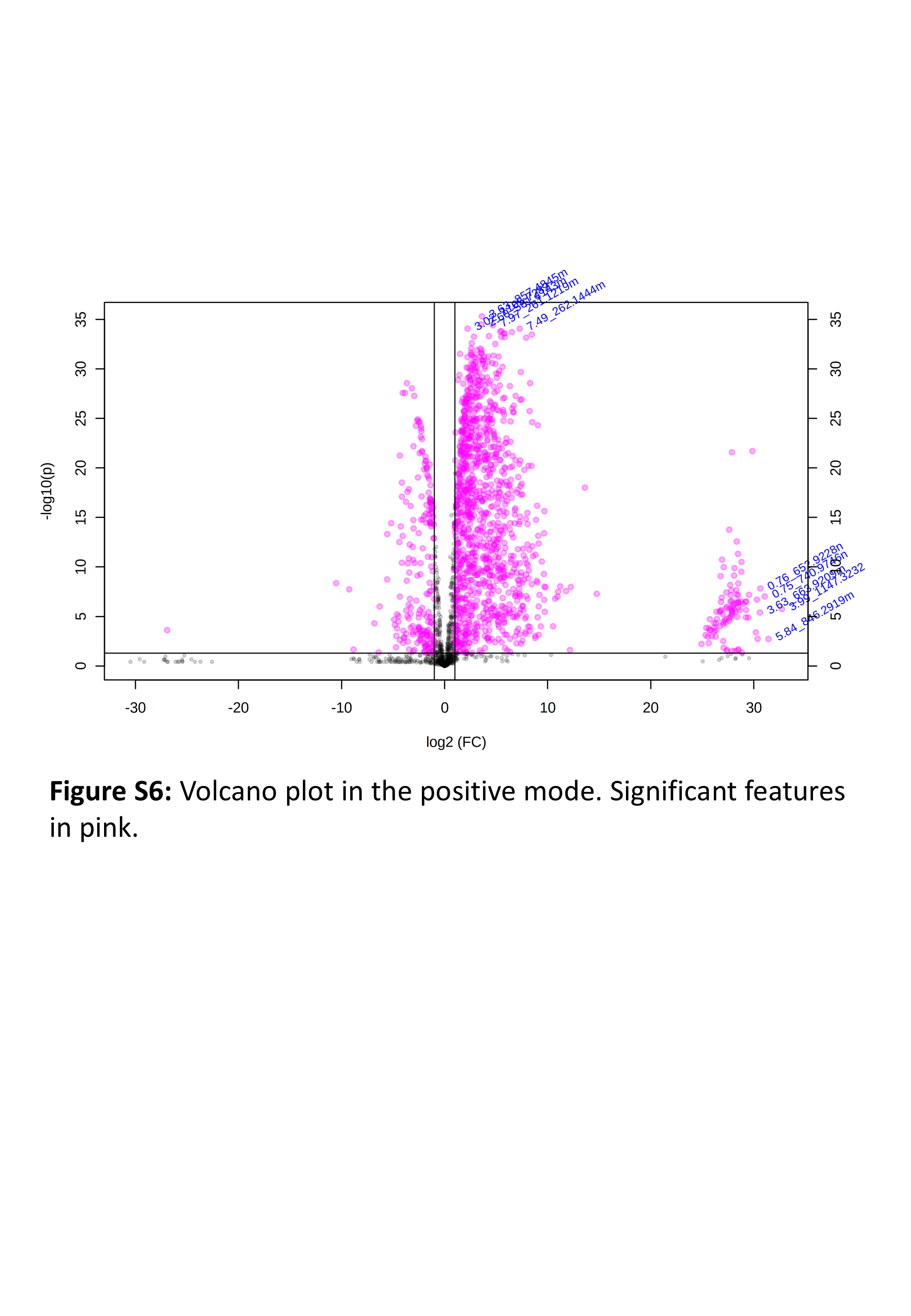

Supplement: Supplementary file 1 [file metabolites-10-00262-s001.zip › Revised_Material-826809/Figure S6_Metabolites.tif]
